# Pandemic (H1N1) 2009 and HIV Infection

**DOI:** 10.3201/eid1706.102018

**Published:** 2011-06

**Authors:** Shireesha Dhanireddy, Robert D. Harrington, Heidi M. Crane, Matthew R. Gingo, Alison Morris, Laurence Huang, Kristina Crothers

**Affiliations:** Author affiliations: University of Washington, Seattle, Washington, USA (S. Dhanireddy, R.D. Harrington, H.M. Crane, K. Crothers);; University of Pittsburgh School of Medicine, Pittsburgh, Pennsylvania, USA (M.R. Gingo, A. Morris);; University of California, San Francisco, California, USA (L. Huang)

**Keywords:** H1N1, influenza, HIV, co-infection, viruses, letter

**To the Editor:** In the United States during spring and fall of 2009, pandemic (H1N1) 2009 influenza A virus resulted in 2 major outbreaks of disease. Initial reports identified immunosuppression, including HIV infection, as a risk factor for the development of severe influenza (*1**–**5*). Subsequent reports did not confirm this association, but the number of HIV-infected patients in these studies was small (*6**,**7*). We describe the clinical course of pandemic (H1N1) 2009 in HIV-infected persons in a US hospital.

During 2009, 23 cases of laboratory-confirmed pandemic (H1N1) 2009 in HIV-infected persons were identified at Harborview Medical Center (Seattle, WA, USA) by querying the University of Washington HIV Information System (a database that enables complete capture of all HIV testing results at Harborview Medical Center) and by querying the Harborview Infection Control Registry for influenza subtype H1N1 infections. Most cases occurred during October and November. Baseline patient characteristics are noted in the Table. Most patients who sought care had fever and cough; median duration of symptoms before seeking care was 4 days. Overall mortality rate for the entire cohort was 8.7%.

**Table:** Baseline characteristics for HIV-infected patients with pandemic (H1N1) 2009, Seattle, Washington, USA, 2009*

Patient characteristics	All patients, n = 23	Inpatients, n = 8	Outpatients, n = 15	p value†
Demographics				
Male sex	19 (83)	5 (63)	14 (93)	0.1
Median age, y (range)	43 (22–72)	46 (34–72)	42 (22–64)	0.5
Race/ethnicity				1.0
White	14 (61)	5 (63)	9 (60)	
Black	5 (22)	2 (25)	3 (20)	
Hispanic	4 (17)	0	4 (27)	1.0
Other/refused to answer	4 (17)	1 (13)	3 (20)	
History				
Received 2009–10 seasonal influenza vaccine before illness	14 (61)	6 (75)	8 (53)	0.1
Ever smoked	15 (65)	7 (88)	8 (53)	0.2
HIV-associated factors				
CD4 count, cells/µL, median (range)	308 (32–1,024)	147 (32–1,024)	438 (119–833)	0.06
Undetectable HIV-1 RNA	19 (83)	6 (75)	13 (87)	0.6
Receiving antiretroviral therapy	21 (91)	8 (100)	13 (87)	0.5
Predisposing risk factors	15 (65)	6 (75)	9 (60)	0.5
Prior lung disease	8 (35)	5 (63)	3 (20)	0.07
Cardiovascular disease	4 (17)	3 (38)	1 (6.7)	0.1
Obesity, body mass index >30	4 (17)	1 (13)	3 (20)	1.0
Neutropenia	3 (13)	2 (25)	1 (6.7)	0.3
Receiving immunosuppressive agent	3 (13)	3 (38)	0	0.03
Malignancy	4 (17)	2 (25)	2 (13)	0.6
Diabetes	2 (8.7)	0	2 (13)	0.5
Signs and symptoms				
Fever	18 (78)	7 (88)	11 (73)	0.6
Fatigue	5 (22)	2 (25)	3 (20)	1.0
Malaise	11 (48)	7 (88)	4 (27)	0.009
Myalgia	10 (43)	3 (38)	7 (47)	1.0
Sore throat	5 (22)	2 (25)	3 (20)	1.0
Cough	21 (91)	8 (100)	13 (87)	0.5
Dyspnea	8 (35)	4 (50)	4 (27)	0.4
Nausea/vomiting	10 (43)	2 (25)	8 (53)	0.4
Median duration of symptoms before seeking care, d (range)	4 (0–30)	4.5 (0–10)	3 (1–30)	0.6
Physical examination findings				
Median temperature, °C (range)	38.0 (35.7–40.2)	39.1 (37.3–40.2)	37.7 (35.7–38.5)	0.001
Median heart rate, beats/min (range)	96 (69–129)	109 (80–127)	94 (69–129)	0.1
Mean arterial blood pressure, mm Hg (range)	94 (66–116)	89 (66–98)	97 (75–116)	0.05
Median respiratory rate, breaths/min (range)	20 (14–40)	22 (18–40)	19 (14–36)	0.04
Abnormal lung sounds	11 (48)	8 (100)	3 (23)	0.001
Laboratory findings				
Leukocyte count, cells × 10^9^/L (range)	4.53 (0.53–10.8)	3.2 (0.53–10.8)	5.4 (2.8–9.4)	0.4
Leukopenia, <5,000 cells/µL	9 (39)	5 (63)	4 (40)	0.6
Chest radiograph findings, new infiltrate	6 (26)	5 (63)	1 (13)	0.1
Care received				
Antiviral treatment for influenza	21 (91)	8 (100)	13 (87)	0.5
Intensive care unit admission	3 (13)	3 (38)	NA	
Mechanical ventilation	2 (8.7)	2 (25)	NA	
Vasopressors	2 (8.7)	2 (25)	NA	
Outcomes				
Secondary pneumonia	3 (13)	3 (38)	0	0.03
Thrombotic complications	1 (4.6)	1 (13)	0	0.4
Died	2 (8.7)	2 (25)	0	0.1

*Values are no. (%) patients except as indicated. Among the 15 outpatients, 13 had a lung examination documented, 10 had a leukocyte count performed, and 8 had a chest radiograph taken. NA, not applicable. †For comparison of characteristics between inpatients and outpatients.

Of the 23 patients, only 2 were not treated for influenza; each had mild signs and symptoms and neither required hospital admission. Each of the remaining 13 outpatients received a 5-day course of treatment with oseltamivir. The 8 patients who required hospitalization received therapy for a median of 6 (range 1–22) days.

Overall mortality rate among HIV-infected patients hospitalized for pandemic (H1N1) 2009 infection was 25% (2 of 8 patients). The 2 inpatients who died had each received >14 days of therapy with oseltamivir. Three inpatients were admitted to the intensive care unit (ICU); of these, 2 had hypoxemic respiratory failure and bilateral infiltrates at the time of admission and a later diagnosis of acute respiratory distress syndrome, and 1 was hospitalized with fever and hemodynamic instability. Each patient with acute respiratory distress syndrome subsequently died; 1 had methicillin-resistant *Staphylococcus aureus* pneumonia at the time of admission, and 1 had severe hypoxemic respiratory failure requiring the use of rescue therapies (e.g., prone positioning and inhaled nitric oxide) and later treatment for ventilator-associated pneumonia. Of the 2 patients who died, 1 had concurrent conditions, including preexisting interstitial lung disease (believed to be associated with crack cocaine use) and a low CD4 cell count of 127 cells/µL, and 1 had a preserved CD4 cell count >1,000 cells/µL, but 8 days passed before anti-influenza therapy was started, and thrombotic complications developed before death. The lengths of ICU stay for the patients who died were 13 and 29 days.

Our findings are similar to those reported by others, suggesting that HIV infection alone does not appear to be a risk factor for severe pandemic (H1N1) 2009, provided that patients are not severely immunocompromised, do not have other risk factors associated with poor outcomes, and are treated for influenza soon after signs and symptoms develop (*6**–**9*). Most of the 23 patients described here had mild disease and were treated as outpatients. Only 3 required ICU admission, and 2 of these died. Although the mortality rate reported here is higher than that reported in other studies, our sample size was relatively small, and the patients who died had additional risk factors for poor outcomes.

Our study has several limitations. It is a retrospective study, and HIV-infected patients at Harborview Medical Center were not all prospectively tested for pandemic (H1N1) 2009. Most pandemic (H1N1) 2009 virus was detected by reverse transcription PCR of nasal swab specimens; this testing was only available after October 2009, during the second wave of influenza. Infections occurring during the spring were diagnosed by insensitive testing with fluorescent antibody and culture, diagnosed by clinical criteria alone and not included in this analysis, or missed altogether.

Because of differences in pandemic (H1N1) 2009 virus testing, we were unable to compare the incidence of pandemic (H1N1) 2009 virus infection and outcomes between HIV-infected and HIV-uninfected patients. A total of 189 persons received a diagnosis of pandemic (H1N1) 2009 at Harborview Medical Center in 2009, and 79 were hospitalized. A total of 8 (10%) of 79 patients with pandemic (H1N1) 2009 died, including the 2 HIV-infected patients reported here. However, during the peak of the epidemic, many HIV-infected outpatients, who were receiving antiretroviral therapy and had preserved CD4 cell counts, were advised to remain at home if they had mild influenza-like symptoms and were therefore not tested for influenza. This circumstance could have produced a bias toward diagnosing and reporting only more severe disease. Outpatients who had influenza-like symptoms were tested and treated empirically pending test results. Our case series of HIV-infected patients with pandemic (H1N1) 2009 at a single institution in the United States suggests that HIV itself does not appear to be as major a risk factor for severe disease as are other previously reported concurrent conditions, delays in treatment, and development of secondary bacterial pneumonia.
